# The Contribution of Sleep Texture in the Characterization of Sleep Apnea

**DOI:** 10.3390/diagnostics13132217

**Published:** 2023-06-29

**Authors:** Carlotta Mutti, Irene Pollara, Anna Abramo, Margherita Soglia, Clara Rapina, Carmela Mastrillo, Francesca Alessandrini, Ivana Rosenzweig, Francesco Rausa, Silvia Pizzarotti, Marcello luigi Salvatelli, Giulia Balella, Liborio Parrino

**Affiliations:** 1 Sleep Disorders Center, Department of Medicine and Surgery, University Hospital of Parma, Via Gramsci 14, 43126 Parma, Italy; carlotta.mutti88@gmail.com (C.M.); irene.pollara@unipr.it (I.P.); aabramo@ao.pr.it (A.A.); margherita.soglia@unipr.it (M.S.); crapina@ao.pr.it (C.R.); cmastrillo@ao.pr.it (C.M.); falessandrini@ao.pr.it (F.A.); francesco.rausa89@gmail.com (F.R.); spizzarotti@ausl.pr.it (S.P.); marcelloluigi.salvatelli@unipr.it (M.l.S.); giulia.balella@unipr.it (G.B.); 2 Sleep Disorders Centre, Guy’s and St Thomas’ NHS Foundation Trust, London SE1 7EH, UK; ivana.1.rosenzweig@kcl.ac.uk; 3 Neurology Unit, Department of Medicine and Surgery, University Hospital of Parma, Via Gramsci 14, 43126 Parma, Italy

**Keywords:** cyclic alternating pattern, sleep texture, sleep apnea, polysomnography

## Abstract

Obstructive sleep apnea (OSA) is multi-faceted world-wide-distributed disorder exerting deep effects on the sleeping brain. In the latest years, strong efforts have been dedicated to finding novel measures assessing the real impact and severity of the pathology, traditionally trivialized by the simplistic apnea/hypopnea index. Due to the unavoidable connection between OSA and sleep, we reviewed the key aspects linking the breathing disorder with sleep pathophysiology, focusing on the role of cyclic alternating pattern (CAP). Sleep structure, reflecting the degree of apnea-induced sleep instability, may provide topical information to stratify OSA severity and foresee some of its dangerous consequences such as excessive daytime sleepiness and cognitive deterioration. Machine learning approaches may reinforce our understanding of this complex multi-level pathology, supporting patients’ phenotypization and easing in a more tailored approach for sleep apnea.

## 1. Introduction 

Sleep-disordered breathing (SDB) defines a heterogeneous group of sleep pathologies encompassing obstructive sleep apnea (OSA), central sleep apnea (CSA), and other rarer sleep-related breathing pathologies. 

OSA is a world-wide-diffused disorder associated with serious multi-systemic consequences. 

It is estimated that around a billion adults suffer from mild-to-severe OSA while more than 400 million adults are affected by moderate-to-severe OSA worldwide [1].

Conventionally, OSA is investigated by means of home-set nocturnal cardio-respiratory recording, and diagnosis is based on the number of respiratory events occurring per hour of sleep, as expressed by the apnea-hypopnea index (AHI). A minimum of 4 recording hours is required to consider the sleep study reliable for diagnosis and, according to the number of respiratory events, the clinical relevance is classified as mild (AHI: 5–14 events/h), moderate (AHI: 15–30 events/h) or severe (AHI > 30 events/h). 

As the condition is typically associated with both diurnal and nocturnal consequences, their assessment is also required, according to current diagnostic criteria. In the International Classification of Sleep Disorders (ICSD-3), sleepiness, fatigue, insomnia, snoring, subjective nocturnal respiratory disturbance and observed apneas, and associated medical or psychiatric disorders support the diagnosis of OSA. 

Notably, the coexistence of other chronic diseases, especially cardiovascular disorders, has been evaluated to assess prognosis, and the novel indicator of C-OSA (comorbid-OSA) has been proposed to ease sleep clinicians in the evaluation of mortality risk in patients with OSA [2]. In a bidirectional perspective, if the concurrence of other disabling pathologies may worsen prognosis, intermittent hypoxia and sleep fragmentation induced by OSA can increase the severity of comorbid cardiovascular and metabolic pathologies [3]. Recently, the severity of OSA-associated sleep fragmentation, measured with the arousal index, has been linked to increased coronary plaque burden, suggesting a direct association between the extent of sleep instability and cardiovascular risk [4]. This observation is important, as routinely PSG is not required for OSA diagnosis, increasing the risk of an under-rating of the cardiovascular impact of the condition. Finally, C-OSA with cardiovascular diseases may coexist with central-type respiratory patterns, such as Cheyne–Stokes breathing (CSB). It has been recently proved that the remote monitoring of CPAP devices in OSA patients may reveal early signs of incipient heart failure, such as long cycles of CSB [5]. The tight association between clinical disorders and sleep apnea suggests that SDB should be investigated in all patients.

## 2. The Limits of AHI

Once sleep apnea has been identified, it is important to measure severity with reliable methods. The predictive value of AHI has been evaluated in a real-world setting, demonstrating a low sensitivity (19%) and a high specificity (84.4%) in the identification of patients affected by mild, moderate or severe OSA [6]. AHI also fails in the identification of OSA patients at a higher risk for diabetes, while the hypoxic burden appears more reliable to predict the metabolic consequences of the disease [7]. Overall, AHI appears as a poor indicator for disease severity and alternative measures are deemed necessary. 

**Figure 1:**
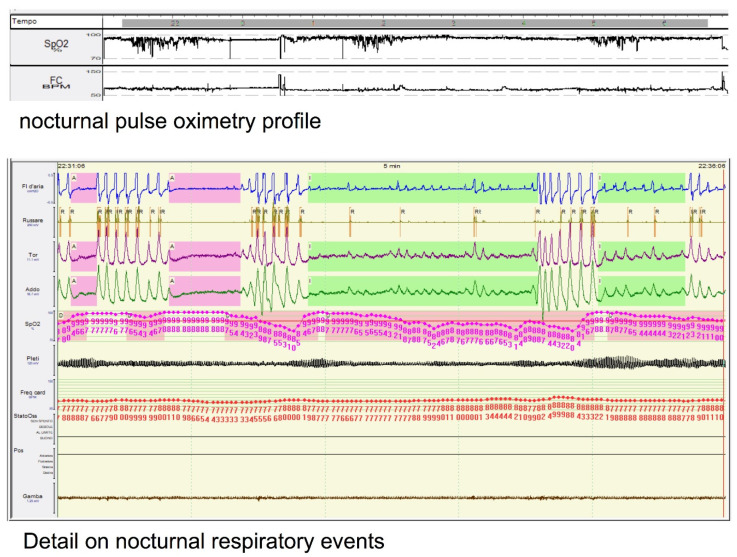
Example of a nocturnal pulse oximetry profile (upper part of the figure), with a detail on her nocturnal respiratory events (lower part of the figure), in a female patient affected by mild OSA (AHI 12.2 events/h). Despite the lower value of AHI, her hypopneas (highlighted in light green) were associated with severe drops in oxygen saturation and respiratory efforts.

In Figure 1 we summarize the polysomnographic data of a 52-year-old obese Italian woman (body mass index 42), with a personal history of arrhythmia and nocturnal sinus pauses, complaining of snoring and non-refreshing sleep. Her cardio-respiratory recording was consistent with a mild OSA (AHI 12.2 events/h). However, looking at the Sat O2 profile and the morphology of her apneas, we could ascertain that the hypoxic load of the patient was compatible with a much more severe clinical condition and thus, regardless of the number of respiratory events measured be the AHI, nocturnal non-invasive ventilation was applied with clinical benefits. 

In the latest years, increasing efforts have been dedicated to the identification of non-AHI-dependent measures for OSA severity, the most significant being the Sleep Revolution Project [8]. Furthermore, current definitions neglect the ‘OSA patients’ pathophysiologic traits’, such as upper airway function, anatomical collapsibility, loop gain, arousal threshold, and sleep structure.

## 3. OSA and Sleep Architecture

Curiously, SDB occurs during sleep, but the recording of brain activity during sleep is not required for diagnosis. This contradiction is due to the high costs and demanding effort to carry out a complete video-polysomnography (PSG) recording, a burden which can be easily bypassed to screen a condition that affects nearly a billion people world-wide [1]. However, it is well known that OSA strongly impacts the structural organization of the sleeping brain. PSG can be analyzed both at macro- and microstructure levels. Sleep macrostructure refers to conventional sleep architecture, which is composed of subsequent 30 s sleep epochs, belonging to different sleep stages, as firstly proposed by Rechtschaffen and Kales back in 1960 [9]. Conversely, sleep microstructure defines all the transients (e.g., sleep spindles, arousals, CAP) appearing during sleep and which are not forcibly confined in the rigid temporal domain of sleep epochs.

Compared to healthy subjects, at the macrostructure level, patients with sleep apnea show reduced amounts of slow-wave sleep (SWS) and increased amounts of lighter sleep stages. Severe OSA also presents a longer latency to stage N3 compared to the mild and moderate subtypes [10].

Interestingly, while a paraphysiological drop in SWS is expected with aging, the effect of sleep apnea on stage N3 reduction appears much stronger [11]. Regardless of the OSA severity, the number of respiratory events in stage N3 is significantly lower compared to the other sleep stages [10]. The lack of respiratory events during SWS probably depends on the higher stability of this sleep stage, which physiologically contains minor amounts of microstructural oscillations [12]. Human NREM sleep is characterized by periodic oscillatory patterns that reflect its inner adaptability and readiness [13]. The cyclic alternating pattern (CAP) is the main representative of NREM microstructural dynamics and is deemed as the electroencephalographic (EEG) hallmark of sleep instability [14]. 

The restorative power of sleep is influenced by the amount of CAP [15], expressed by repetitive cycles composed of a phase A (activation) and its subsequent phase B (de-activation). CAP, by nature, overcomes the rigid boundaries of 30 sec of duration for sleep epochs and elevates the sleep scoring to a more realistic and dynamic framework. Each phase of the CAP cycle can range from 2 to 60 s in duration. At least two CAP cycles are required to identify a CAP sequence. The amount of CAP oscillations during NREM sleep is measured by CAP rate. CAP is observed as sequences of transient electrocortical events which appear distinct from the ongoing tonic background and recur at periodic intervals. CAP phase A can be composed of different electroencephalographic patterns including delta bursts, vertex sharp transients, K-complex sequences, polyphasic bursts, k-alpha, intermittent alpha, or typical EEG arousals.

**Figure 2:**
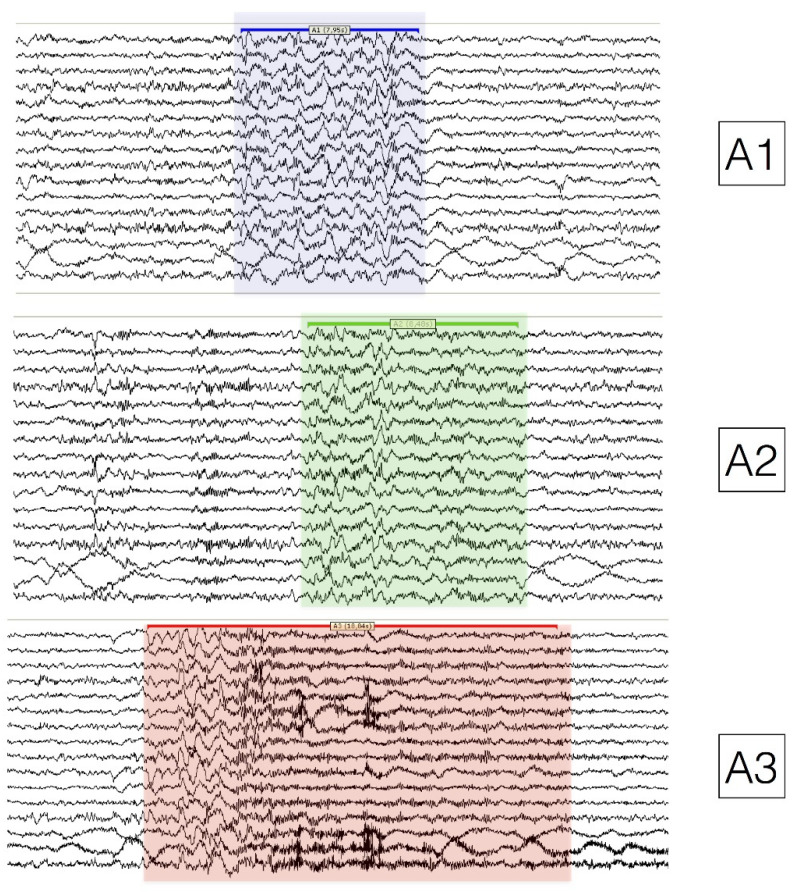
Polysomnographic example of CAP phase A subtypes. CAP (**A1**) subtype is highlighted in light blue, (**A2**) in light green, and (**A3**) in light red.

According to the EEG features, CAP phase A can be further classified as subtypes A1, A2, and A3 (see Figure 2). In the A1 subtype, the EEG synchrony (high-amplitude slow waves) is the predominant activity and, if present, EEG desynchrony (low-amplitude fast waves) occupies less than 20% of the entire phase A duration. The phase A2 subtype is characterized by a mixture of slow and fast rhythms with 20% to 50% of phase A occupied by EEG desynchrony. Finally, CAP phase A3 is dominated by rapid low-voltage rhythms with more than 50% of phase A expressed by EEG desynchrony [16]. The distinction between phase A subtypes is not trivial as they have different roles in sleep organization. Subtype A1 prevails in the first half of the night and boosts the build-up and consolidation of SWS, while subtypes A2 and A3 increase in the second part of the night and prepare the sleeping brain for the appearance of REM sleep or wakefulness. Furthermore, subtype A3 exerts a stronger impact on cardiovascular parameters compared to subtypes A1 and A2 [17]. Overall, CAP metrics quantify the magnitude of stressful perturbation on the sleeping brain and its kinetics strongly correlate with the autonomic and behavioral function at whole-body level [18].

CAP also reflects the specific impact of diseases on sleep organization, providing additional information compared to the American Academy of Sleep Medicine (AASM) arousal rules in OSA patients; the physiological behavior of sleep instability (involving both respiratory events and EEG activation) overcomes the limited quantification of conventional ‘fast arousals’ [19]. 

Accordingly, sleep instability, which represents one of the main factors responsible for excessive daytime sleepiness in OSA patients, cannot be measured only with the count of AASM conventional arousals, but should also include the (commonly neglected) ’subcortical arousals’, which are frequently accompanied by various degrees of autonomic stimulation [20]. In this perspective, sleep microstructure may be highly informative. In the clinical domain, CAP subtype A1 prevails in the milder OSA phenotype, whilst subtypes A2 and A3 abound in patients with moderate-to-severe OSA [21]. Notably, a higher amount of sleep fragmentation and lower representation of SWS has also been noticed in patients affected by Upper Airway Resistance Syndrome (UARS), a condition firstly described in 1993 and nowadays considered as the milder phenotype of OSA. Patients affected by UARS typically complain of excessive daytime sleepiness, non-refreshing sleep, and might develop various daytime consequences (such as mood disturbances and/or cognitive impairment). Sleep instability in patients with UARS has traditionally been linked to excessive respiratory effort and flow limitation [22]. Notably, compared to the conventional AASM sleep scoring system, sleep microstructure and CAP ease the detection of sleep fragmentation in patients suffering from UARS, which appears strongly correlated with subjective symptoms (daytime fatigue, sleepiness) [23].

If the CAP system still promotes sleep continuity in OSA milder cases, the situation becomes unbalanced in moderate–severe OSA, where the CAP system turns into an intrusive phenomenon, perturbing sleep dynamics [21]. The existence of two distinct arousal-related mechanisms in human sleep is similar to what has been described in rodents [24]. According to this model, depending on the stimulated regions of the parabrachial nucleus (PBN), arousal may yield to either fast-frequency bursts, strongly similar to A2/A3 CAP subtypes (when the more lateral regions of the PBN are stimulated) or high-amplitude slow-waves, in response to the medial PBN stimulation [25]. In this perspective, CAP can phenotype the ‘arousal-gate’ in patients with sleep apnea. 

Sleep microstructure can also inform on the neurobehavioral adaptability to sleep fragmentation in OSA patients. In fact, it has been suggested that the AHI is inconsistently associated with behavioral performances in patients affected by sleep apnea: while some patients may be highly vulnerable to sleep loss, some others can be impressively resistant to the negative effects of sleep deprivation [26]. This is not trivial as, for instance, individual resilience to sleep loss might influence driving performance and subsequent risk for car crashes [26]. While neither conventional polysomnographic metrics nor the AASM arousal index were predictive of neurobehavioral impairment, it has been documented that higher EEG slowing ratios during REM and lower spindle density at the frontal EEG were significantly associated with slower reaction times in a psychomotor vigilance task and driving simulator test [27]. In parallel, it has been demonstrated that, in ventilated OSA patients, the utilization of suboptimal continuous positive airway pressure (CPAP) may favor the recurrence of sustained inspiratory airflow limitation, which in turn is associated with abnormal K-complexes (coupled with bursts of alpha as arousal surrogates). Hence, suboptimal CPAP therapy can lead to subtle/‘nonvisible’ sleep fragmentation [28].

Another interesting measure adopted to indicate sleep continuity is represented by the odds ratio products (ORPs) [29]. The ORPs represent an attempt to capture the transitions within sleep/wakefulness states during clinical polysomnography, in order to overcome the rigid subdivisions imposed by the conventional Rechtschaffen and Kales rules for PSG scoring. ORPs correlate with sleep depth and quality, and this metric is calculated as a ratio of the absolute power of different frequency bands over 3 s segments. The ORP ratio ranges from 0 to 2.5, with 0 being indicative of very deep sleep and 2.5 being related to a wide-awake state [29]. ORPs might serve as novel biomarkers for PSG in various sleep conditions. In OSA, for instance, it has been proved that, compared to healthy sleepers, ORPs are usually lower during an awake state (suggesting increased sleep propensity) but NREM is higher (consistent with lighter NREM sleep) [30]. Among the advantages of using ORPs, there is the possibility to verify the arousal effects towards sleep depth, which can be inferred by the speed with which sleep deepens following an arousal, to so-called postarousal sleep dynamics [31]. When ORPs are high, arousal stimuli can easily trigger arousal, whereas when ORPs are low, strong stimuli are required to provoke an arousal. In other words, the metric of ORPs informs us of the extent to which patients can recover sleep after arousal intrusions [31]. 

Although the disorder is typically evaluated using cardio-respiratory recording, some informative features can be inferred from the ongoing brain activity: for instance, the pulse wave amplitude has been associated with the amount of EEG response (including CAP features) to respiratory events, suggesting a strong coupling between cortical and autonomic activation [32].

## 4. Breathing Oscillations, Daytime Sleepiness and Treatment Outcomes

The temporal association between CAP oscillations and respiratory events is predictable: in fact, most apneas/hyponeas co-occur with CAP phase B and thus the disease is frequently defined as a prototypical example of a CAP phase-B disorder [33]. As a translation of the lesser arousal branch of arousal swings, phase B represents a highly vulnerable background for upper airway collapse and for attenuation of biochemical and neural mechanisms in the control of the ventilatory drive, while phase A is mandatory to restore breathing. It has been demonstrated that the vast majority of respiratory events are coupled with CAP (96% in NREM and 80% in REM sleep). In detail, the apneas and hypopneas are commonly temporally associated with CAP phase B, while breathing restoration is typically recovered during phase A [34]. Recently, a large study analyzing over 1.6 million cortical arousals, 350,000 apneas, and over 1.9 million hypopneas collected from 11,400 manually scored PSG recordings available online demonstrated that the duration of 95–99% of all respiratory events falls beneath 60 s [35]. This finding is supported by a solid physiological background as the length of CAP phase B (which triggers and supports breathing interruption) spans within the 1 min interval [15].

CAP metrics correlate with subjectively perceived daytime sleepiness in patients with OSA [36]. In male subjects with moderate-to-severe OSA, CAP rate, CAP duration, the number of CAP cycles, and the duration and percentage of the subtype A2 are significantly higher in patients with EDS compared to those without EDS. In contrast, the two groups (EDS vs. non-EDS) showed no differences in conventional sleep parameters. In other words, higher levels of arousal activation induced by respiratory events determine a more aggressive sleep fragmentation which leads to a more somnolent daytime setting.

**Figure 3:**
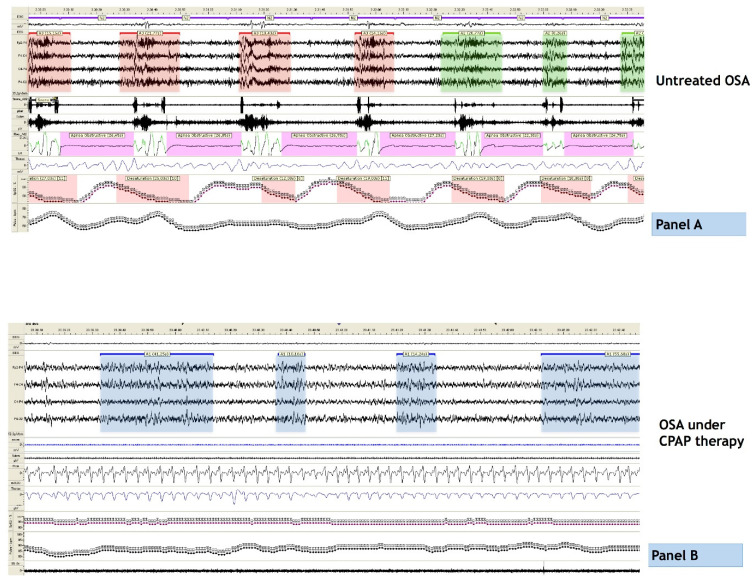
Polysomnographic example of a patient affected by severe OSA before (**A**) and after (**B**) the introduction of CPAP. Panel A: note the tight association between apneas and cyclic alternating pattern (CAP) oscillations, with strong prevalence of subtypes A2 and A3, with high autonomic impact; panel B: observe the restoration of physiologic CAP oscillations, with predominance of subtype A1. In details, at PSG level, light red highlight CAP phase A3 subtypes, light green refer to A2 subtypes and light blue to A1 subtypes.

PSG can also be used to assess treatment benefits on patients with severe OSA. It has been proved that continuous positive airway pressure (CPAP), the first-line treatment for moderate-to-severe OSA, increases the duration of N3 and decreases the lightest NREM sleep stages. With respect to microstructural PSG parameters, the reduction in respiratory events promotes an attenuation of CAP fluctuations, in particular during stage N3, and a parallel increase in the CAP oscillations that occur independent from apneas/hypopneas [37]. In this perspective, we can summarize that CPAP promotes the restoration of a more physiological CAP oscillatory pattern, with oscillations that are disengaged from breathing constraints. Interestingly, the timing of CPAP-dependent PSG variations is not the same for all sleep features: for instance, CPAP treatment induces an immediate restoration of sleep continuity, a more consolidated REM sleep, a curtailment of sleep latency, and an enhancement of SWS, while the amount of CAP cycles and A1 subtypes remains below normal values even one month after the introduction of ventilatory support [38] (Figure 3). An impressive increase in the SWS occurs already during the first night of CPAP, together with a parallel normalization in the overall duration of CAP phases A and B, both significantly longer in patients with severe OSA [39]. Furthermore, in patients with severe OSA treated with CPAP, variations of CAP rate significantly correlate with daytime vigilance [39]. 

Sleep recordings may also provide information on the impairment of cognitive processes, a detrimental consequence in patients affected by untreated OSA [40,41]. The extent of cognitive impairment, assessed with the Montreal Cognitive Assessment (MoCA), has been found to be related to the severity of sleep fragmentation as expressed by the CAP A3 rate in patients with sleep apnea [42]. A similar association between CAP metrics and cognitive deterioration has been described by Karimzadeh et al. [43], who demonstrated a direct correlation between the quantity of CAP phase A1 fluctuations and cognitive performances, especially in verbal fluency, memory, and visuo-spatial skills, in patients affected by OSA. Patients suffering from sleep apnea presented a higher density of CAP phase A oscillations within the fronto-parietal regions, a condition that may interfere with NREM sleep-dependent cognitive processes. Recently, impaired social cognition has also been identified in middle-aged patients affected by OSA with no other relevant neurological or psychiatric conditions, potentially associated with the pathology-dependent impairment of the REM sleep stage [40].

The application of CAP scoring can support the phenotyping of various diseases as demonstrated for Down syndrome, Fragile-X syndrome, and attention deficit hyperactivity disorder, where the evaluation of sleep microstructure can help in the identification of patients suffering between ‘primary disease forms’ (lower amounts of CAP oscillations) from those with coexistent sleep-related breathing disorders (higher CAP rate values) [44,45]. In the latest years, the European Sleep Apnea Database (ESADA) has tried to detail OSA heterogeneity using clustering procedures. The OSA clusters identified from the ESADA consortium are based on gender, age, symptoms, comorbidities, and respiratory features, but so far, no conventional PSG measures have been included. The possibility to identify OSA phenotypes might improve patients’ prognostication and ease the identification of targeted therapies. From an operational point of view, ‘phenotypes’ can be described as ‘*A category of patients with OSA distinguished from others by a single or combination of disease features, in relation to clinically meaningful attributes (symptoms, response to therapy, health outcomes, quality of life*’. The possibility to reveal individual differences through phenotypes is fundamental to tailor treatment prescription and to increase long-term compliance [46]. For instance, it has been demonstrated that patients with REM-dominant OSA present reduced total sleep time, decreased sleep efficiency, and lack of REM sleep representation [47]. REM-dominant OSA patients also present reduced response to mandibular advancement splints [48] and, due to the association between REM-related AHI indices and incident hypertension, they likely deserve non-invasive ventilation, which should be guaranteed in the second half of the night, when REM sleep is largely prevalent [49,50]. Conversely, patients with NREM-dominant OSA present higher ventilator control instability during NREM sleep (which paradoxically improves during REM sleep), a condition that may coexist with a low arousal threshold and which might benefit from pharmacological sleep-stabilizing approaches [51,52,53]. 

In this perspective, the utilization of polysomnographic features (with combined macro and microstructure analysis) may reinforce the reliability of any attempt of OSA phenotypization, leading to strongly predictive approaches, with potential clinical implications.

## 5. The Contribution of Machine Learning

In order to curtail a timely OSA diagnosis, a number of portable/wearable hardware devices, based on machine learning approaches, have been proposed to screen for this condition. Their lower costs and the ease of use compared to conventional polysomnography stand out among the major advantages. However, the absence of medical supervision may expose patients to the risk of less accurate diagnosis. Most of these devices work on photoplethysmographic (PPG) sensors to detect oxygen desaturations. In this case, the information regarding breathing events is indirectly collected from blood flow patterns in the microvascular tissue bed [54]. Although it is still insufficient to ascertain a diagnosis, the utilization of wearable devices has gained increasing importance in the screening of subjects with a high risk for OSA [55]. Artificial intelligence can be adopted to evaluate polysomnographic findings in order to screen for sleep apnea. The automated classification of polysomnographic features (including macro- and microstructure data) has already been tested for various sleep pathologies including narcolepsy, rapid eye movement behavior disorder, periodic leg movement disorder, sleep-related epilepsy, and insomnia [56,57]. Considering the strong impact of OSA on sleep organization, the availability of automated methods to score sleep recordings might ease the identification of clusters of electrophysiological data, potentially related to distinct clinical phenotypes. In this perspective, machine learning could represent a relevant step forward in the intricate scenario of sleep apnea diagnosis and phenotypization. So far, the most important applications of artificial intelligence in obstructive sleep apnea have been the following: (1) predicting treatment outcomes of various treatment options, (2) improving treatment options, and (3) personalizing treatment thanks to the enhanced understanding of underlying mechanisms of the disease [58]. This new ‘personalized’ approach to sleep apnea may increase patients’ compliance and increase the efficacy of proposed approaches. The utility of machine learning has also been suggested to select patients assigned to CPAP therapy, surgical treatment, and/or oral appliances. The possibility to predict therapeutic success with non-invasive techniques represents an ambitious goal for sleep specialists. Due to unavoidable links between the autonomic system and electroencephalographic findings, few authors explored the bidirectional association between cardiopulmonary functioning and EEG signals. Simplifying all the NREM sleep stages into two opposite dimensions, i.e., stable versus unstable NREM sleep, it has been proved that stable breathing periods result in high-frequency coupling of respiration and HRV, with the simultaneous presence of higher EEG delta power. In parallel, the so-called unstable NREM sleep may be recognized by a sleep fragmentation phenotype [59]. Sleep apneas are strongly associated with cardiovascular consequences and autonomic imbalance. The periodic alterations in sympathetic nervous activity and parasympathetic nerve activity during each respiratory event define a peculiar heart rate pattern, which seems like a signature of the condition. Typically, we can notice a drop in heart rate, which is a physiological reaction to the apnea (known as the ‘diving reflex’), immediately followed by a phasic acceleration in heart rate (the relative tachycardia), restoring blood–gas exchange in the lung. This alternation can also be schematized in terms of periodic shifts between parasympathetic and sympathetic tone dominance. Given the periodic reproducibility of these cardiological variations, mathematical approaches and machine learning techniques have been adopted to assess their dynamics in sleep apnea. However, due to inter- and intra-individual variability of cardiovascular activity related to physical training conditions, age, weight, and concomitant diseases, the utilization of the sole cardiac signal is still largely insufficient to represent OSA severity [60,61]. In conclusion, automated sleep scoring approaches will probably guarantee a simplified approach to sleep microstructure analysis that, hopefully, will be followed by world-wide diffusion of its application in everyday sleep scoring practice. This might also promote a higher homogeneity in PSG microstructure scoring, favoring a more reliable comparability between study results. However, due to the inner complexity of human sleep, we believe that, even with the most sophisticated techniques, the role of the ‘man-in-loop’, supervising the entire process, should never be neglected. 

## 6. Conclusions

In the present contribution, we summarized the informative value of sleep recording in the diagnosis and management of OSA, with a focus on sleep microstructure. Although not commonly evaluated in the routine OSA work-up, we remarked on the key role of sleep assessment to deeply understand the disease severity at an individual level. While international guidelines promote the ‘one-size-fits-all’ strategy for OSA management, it is important to remember that the disease may present important inter-individual differences, reasonably warranting a more tailored approach. In this framework, video-PSG and sleep microstructure could provide additional information in the clinical management of patients with SDB. In addition, CAP-related arousal instability seems to be one of the key mechanisms explaining cognitive decline and vigilance impairment in sleep apnea. The cost and complexity of CAP scoring can be overcome by the availability of reliable automatic scoring systems, which will help both researchers and clinicians to better define the impact of SDB on the sleep texture as well as neurological and extra-neurological consequences. The vertical integration of combined approaches might ease our understanding of this complex sleep pathology. 

## Data Availability

Not applicable.

## References

[1] Benjafield A.V., Ayas N.T., Eastwood P.R., Heinzer R., Ip M.S.M., Morrell M.J., Nunez C.M., Patel S.R., Penzel T., Pépin J.-L. (2019). Estimation of the global prevalence and burden of obstructive sleep apnoea: A literature-based analysis. Lancet Respir. Med..

[2] Chiang C.-L., Chen Y.-T., Wang K.-L., Su V.Y.-F., Wu L.-A., Perng D.-W., Chang S.-C., Chen Y.-M., Chen T.-J., Chou K.-T. (2017). Comorbidities and risk of mortality in patients with sleep apnea. Ann. Med..

[3] Dewan N.A., Nieto F.J., Somers V.K. (2015). Intermittent hypoxemia and OSA: Implications for comorbidities. Chest.

[4] Lu M., Yu W., Wang Z., Huang Z. (2022). Association between Arousals during Sleep and Subclinical Coronary Atherosclerosis in Patients with Obstructive Sleep Apnea. Brain Sci..

[5] Saito K., Takamatsu Y. (2022). Cheyne-Stokes Breathing as a Predictive Indicator of Heart Failure in Patients with Obstructive Sleep Apnea; A Retrospective Case Control Study Using Continuous Positive Airway Pressure Remote Monitoring Data. Front. Cardiovasc. Med..

[6] Silva G.E., Rojo-Wissar D.M., Quan S.F., Haynes P.L. (2021). Predictive ability of the International Classification of Sleep Disorders-3 in identifying risk of obstructive sleep apnea among recently unemployed adults. Sleep Breath..

[7] Wojeck B.S., Inzucchi S.E., Qin L., Yaggi H.K. (2023). Polysomnographic predictors of incident diabetes and pre-diabetes: An analysis of the DREAM study. J. Clin. Sleep Med..

[8] Arnardottir E.S., Islind A.S., Óskarsdóttir M., Ólafsdóttir K.A., August E., Jónasdóttir L., Hrubos-Strøm H., Saavedra J.M., Grote L., Hedner J. (2022). The Sleep Revolution project: The concept and objectives. J. Sleep Res..

[9] Rechtschaffen A., Kales A., National Institutes of Health Publications (1968). A Manual of Standardized Terminology, Techniques and Scoring System for Sleep Stages in Human Subjects.

[10] Wu B., Cai J., Yao Y., Pan Y., Pan L., Zhang L., Sun Y. (2020). Relationship between sleep architecture and severity of obstructive sleep apnea. Zhejiang Da Xue Xue Bao Yi Xue Ban.

[11] Guo D., Peng H., Feng Y., Li D., Xu T., Li T., Liao S. (2015). Effects of obstructive sleep apnea-hypopnea syndrome and age on sleep architecture. Nan Fang Yi Ke Da Xue Xue Bao.

[12] Terzano M.G., Parrino L., Spaggiari M.C. (1988). The cyclic alternating pattern sequences in the dynamic organization of sleep. Electroencephalogr. Clin. Neurophysiol..

[13] Halász P., Bodizs R., Parrino L., Terzano M. (2014). Two features of sleep slow waves: Homeostatic and reactive aspects—From long term to instant sleep homeostasis. Sleep Med..

[14] Parrino L., Ferri R., Bruni O., Terzano M.G. (2012). Cyclic alternating pattern (CAP): The marker of sleep instability. Sleep Med. Rev..

[15] Parrino L., Grassi A., Milioli G. (2014). Cyclic alternating pattern in polysomnography: What is it and what does it mean?. Curr. Opin. Pulm. Med..

[16] Terzano M.G., Parrino L., Sherieri A., Chervin R., Chokroverty S., Guilleminault C., Hirshkowitz M., Mahowald M., Moldofsky H., Rosa A. (2001). Atlas, rules, and recording techniques for the scoring of cyclic alternating pattern (CAP) in human sleep. Sleep Med..

[17] Hartmann S., Ferri R., Bruni O., Baumert M. (2021). Causality of cortical and cardiovascular activity during cyclic alternating pattern in non-rapid eye movement sleep. Philos. Trans. R. Soc. A Math. Phys. Eng. Sci..

[18] Mutti C., Azzi N., Halasz P., Szucs A., Parrino L. (2021). Intra period CAP kinetics to stressful perturbation: A message from obstructive sleep apnea. Sleep Med..

[19] Bosi M., Grassi A., Milioli G., Riccardi S., Terzano M.G., Cortelli P., Poletti V., Parrino L. (2015). Can Sleep microstructure improve diagnosis of OSAS? Integrative information from CAP parameters. Arch. Ital. Biol..

[20] Punjabi N.M., Lim D. (2021). Reply to Dr. Gold’s commentary con: Sleep fragmentation causes hypersomnolence in OSA. Sleep Med. Rev..

[21] Gnoni V., Drakatos P., Higgins S., Duncan I., Wasserman D., Kabiljo R., Mutti C., Halasz P., Goadsby P.J., Leschziner G.D. (2021). Cyclic alternating pattern in obstructive sleep apnea: A preliminary study. J. Sleep Res..

[22] Exar E.N., Collop N.A. (1999). The Upper Airway Resistance Syndrome. Chest.

[23] Guilleminault C., Lopes M.C., Hagen C.C., Rosa A. (2007). The Cyclic Alternating Pattern Demonstrates Increased Sleep Instability and Correlates with Fatigue and Sleepiness in Adults with Upper Airway Resistance Syndrome. Sleep.

[24] Anaclet C., Ferrari L., Arrigoni E., Bass C.E., Saper C.B., Lu J., Fuller P.M. (2014). The GABAergic parafacial zone is a medullary slow wave sleep–promoting center. Nat. Neurosci..

[25] Kaur S., Saper C.B. (2019). Neural circuitry underlying waking up to hypercapnia. Front. Neurosci..

[26] Vakulin A., Catcheside P., Baulk S.D., Antic N.A., Banks S., Dorrian J., McEvoy D. (2014). Individual Variability and Predictors of Driving Simulator Impairment in Patients with Obstructive Sleep Apnea. J. Clin. Sleep Med..

[27] Parekh A., Mullins A.E., Kam K., Varga A.W., Rapoport D.M., Ayappa I. (2019). Slow-wave activity surrounding stage N2 K-complexes and daytime function measured by psychomotor vigilance test in obstructive sleep apnea. Sleep.

[28] Parekh A., Kam K., Mullins A.E., Castillo B., Berkalieva A., Mazumdar M., Varga A.W., Eckert D.J., Rapoport D.M., Ayappa I. (2021). Altered K-complex morphology during sustained inspiratory airflow limitation is associated with next-day lapses in vigilance in obstructive sleep apnea. Sleep.

[29] Younes M., Ostrowski M., Soiferman M., Younes H., Younes M., Raneri J., Hanly P. (2015). Odds Ratio Product of Sleep EEG as a Continuous Measure of Sleep State. Sleep.

[30] Younes M., Azarbarzin A., Reid M., Mazzotti D.R., Redline S. (2021). Characteristics and reproducibility of novel sleep EEG biomarkers and their variation with sleep apnea and insomnia in a large community-based cohort. Sleep.

[31] Younes M., Hanly P.J. (2016). Immediate postarousal sleep dynamics: An important determinant of sleep stability in obstructive sleep apnea. J. Appl. Physiol..

[32] Bosi M., Milioli G., Riccardi S., Melpignano A., Vaudano A.E., Cortelli P., Poletti V., Parrino L. (2018). Arousal responses to respiratory events during sleep: The role of pulse wave amplitude. J. Sleep Res..

[33] Parrino L., Rausa F., Azzi N., Pollara I., Mutti C. (2021). Cyclic alternating patterns and arousals: What is relevant in obstructive sleep apnea? In Memoriam Mario Giovanni Terzano. Curr. Opin. Pulm. Med..

[34] Terzano M.G., Parrino L., Boselli M., Spaggiari M.C., Di Giovanni G. (1996). Polysomnographic Analysis of Arousal Responses in Obstructive Sleep Apnea Syndrome by Means of the Cyclic Alternating Pattern. J. Clin. Neurophysiol..

[35] Zitting K.-M., Lockyer B.B.J., Azarbarzin A., Sands S.A., Wang W., Wellman A., Quan S.F. (2023). Association of cortical arousals with sleep-disordered breathing events. J. Clin. Sleep Med..

[36] Korkmaz S., Bilecenoglu N.T., Aksu M., Yoldas T.K. (2018). Cyclic Alternating Pattern in Obstructive Sleep Apnea Patients with versus without Excessive Sleepiness. Sleep Disord..

[37] Chen S., Li Q., Zou X., Zhong Z., Ouyang Q., Wang M., Luo Y., Yao D. (2022). Effects of CPAP Treatment on Electroencephalographic Activity in Patients with Obstructive Sleep Apnea Syndrome during Deep Sleep with Consideration of Cyclic Alternating Pattern. Nat. Sci. Sleep.

[38] Parrino L., Thomas R.J., Smerieri A., Spaggiari M.C., Del Felice A., Terzano M.G. (2005). Reorganization of sleep patterns in severe OSAS under prolonged CPAP treatment. Clin. Neurophysiol..

[39] Parrino L., Smerieri A., Boselli M., Spaggiari M.C., Terzano M.G. (2000). Sleep reactivity during acute nasal CPAP in obstructive sleep apnea syndrome. Neurology.

[40] Gnoni V., Mesquita M., O’Regan D., Delogu A., Chakalov I., Antal A., Young A.H., Bucks R.S., Jackson M.L., Rosenzweig I. (2023). Distinct cognitive changes in male patients with obstructive sleep apnoea without co-morbidities. Front. Sleep.

[41] Gu Y., Gagnon J., Kaminska M. (2023). Sleep electroencephalography biomarkers of cognition in obstructive sleep apnea. J. Sleep Res..

[42] Li N., Wang J., Wang D., Wang Q., Han F., Jyothi K., Chen R. (2019). Correlation of sleep microstructure with daytime sleepiness and cognitive function in young and middle-aged adults with obstructive sleep apnea syndrome. Eur. Arch. Oto-Rhino-Laryngol..

[43] Karimzadeh F., Nami M., Boostani R. (2017). Sleep microstructure dynamics and neurocognitive performance in obstructive sleep apnea syndrome patients. J. Integr. Neurosci..

[44] Miano S., Castelnovo A., Bruni O., Manconi M. (2021). Sleep microstructure in attention deficit hyperactivity disorder according to the underlying sleep phenotypes. J. Sleep Res..

[45] Miano S., Bruni O., Elia M., Scifo L., Smerieri A., Trovato A., Verrillo E., Terzano M.G., Ferri R. (2008). Sleep phenotypes of intellectual disability: A polysomnographic evaluation in subjects with Down syndrome and Fragile-X syndrome. Clin. Neurophysiol..

[46] Bailly S., Grote L., Hedner J., Schiza S., McNicholas W.T., Basoglu O.K., Lombardi C., Dogas Z., Roisman G., Pataka A. (2021). Clusters of sleep apnoea phenotypes: A large pan-European study from the European Sleep Apnoea Database (ESADA). Respirology.

[47] Zinchuk A.V., Gentry M.J., Concato J., Yaggi H.K. (2017). Phenotypes in obstructive sleep apnea: A definition, examples and evolution of approaches. Sleep Med. Rev..

[48] Sutherland K., Takaya H., Qian J., Petocz P., Ng A.T., Cistulli P.A. (2015). Oral Appliance Treatment Response and Polysomnographic Phenotypes of Obstructive Sleep Apnea. J. Clin. Sleep Med..

[49] Appleton S.L., Vakulin A., Martin S.A., Lang C.J., Wittert G.A., Taylor A.W., McEvoy R.D., Antic N.A., Catcheside P.G., Adams R.J. (2016). Hypertension Is Associated with Undiagnosed OSA During Rapid Eye Movement Sleep. Chest.

[50] Aurora R.N., Crainiceanu C., Gottlieb D.J., Kim J.S., Punjabi N.M. (2018). Obstructive Sleep Apnea during REM Sleep and Cardiovascular Disease. Am. J. Respir. Crit. Care Med..

[51] Joosten S.A., Landry S.A., Wong A.-M., Mann D.L., Terrill P.I., Sands S.A., Turton A., Beatty C., Thomson L., Hamilton G.S. (2021). Assessing the Physiologic Endotypes Responsible for REM- and NREM-Based OSA. Chest.

[52] Thomas R.J., Terzano M.G., Parrino L., Weiss J.W. (2004). Obstructive Sleep-Disordered Breathing with a Dominant Cyclic Alternating Pattern—A Recognizable Polysomnographic Variant with Practical Clinical Implications. Sleep.

[53] Smales E.T., Edwards B.A., Deyoung P.N., McSharry D.G., Wellman A., Velasquez A., Owens R., Orr J.E., Malhotra A. (2015). Trazodone Effects on Obstructive Sleep Apnea and Non-REM Arousal Threshold. Ann. Am. Thorac. Soc..

[54] Puranik S., Morales A.W. (2019). Heart Rate Estimation of PPG Signals with Simultaneous Accelerometry Using Adaptive Neural Network Filtering. IEEE Trans. Consum. Electron..

[55] Khor Y.H., Khung S.-W., Ruehland W.R., Jiao Y., Lew J., Munsif M., Ng Y., Ridgers A., Schulte M., Seow D. (2023). Portable evaluation of obstructive sleep apnea in adults: A systematic review. Sleep Med. Rev..

[56] Murarka S., Wadichar A., Bhurane A., Sharma M., Acharya U.R. (2022). Automated classification of cyclic alternating pattern sleep phases in healthy and sleep-disordered subjects using convolutional neural network. Comput. Biol. Med..

[57] Sharma M., Patel V., Tiwari J., Acharya U.R. (2021). Automated Characterization of Cyclic Alternating Pattern Using Wavelet-Based Features and Ensemble Learning Techniques with EEG Signals. Diagnostics.

[58] Brennan H.L., Kirby S.D. (2023). The role of artificial intelligence in the treatment of obstructive sleep apnea. J. Otolaryngol.-Head Neck Surg..

[59] Parrino L., Halasz P., Szucs A., Thomas R.J., Azzi N., Rausa F., Pizzarotti S., Zilioli A., Misirocchi F., Mutti C. (2022). Sleep medicine: Practice, challenges and new frontiers. Front. Neurol..

[60] Penzel T., Kantelhardt J.W., Bartsch R.P., Riedl M., Kraemer J.F., Wessel N., Garcia C., Glos M., Fietze I., Schöbel C. (2016). Modulations of Heart Rate, ECG, and Cardio-Respiratory Coupling Observed in Polysomnography. Front. Physiol..

[61] Parrino L. (2023). Now that automatic processing makes CAP scoring fast and reliable is the sleep field ready for a paradigm shift?. Sleep.

